# Call to action for equitable access to human milk for vulnerable infants

**DOI:** 10.1016/S2214-109X(19)30402-4

**Published:** 2019-11-01

**Authors:** Kiersten Israel-Ballard, Jessica Cohen, Kimberly Mansen, Michael Parker, Cyril Engmann, Maureen Kelley, for the Oxford-PATH Human Milk Working Group

**Affiliations:** University of Washington, Seattle 98195, WA, USA; PATH, Seattle, WA, USA; PATH, Seattle, WA, USA; Ethox Centre/Wellcome Centre for Ethics and Humanities, University of Oxford, Oxford, UK; University of Washington, Seattle 98195, WA, USA; PATH, Seattle, WA, USA; Ethox Centre/Wellcome Centre for Ethics and Humanities, University of Oxford, Oxford, UK

Breastfeeding, which is an integral component of human reproduction, prevents the infant mortality associated with alternative feeding methods owing to the unique nutritional and immunoactive properties of human milk.^1^ UNICEF and WHO emphasise the crucial role of breastfeeding in addressing the Sustainable Development Goals (SDGs), including improving child nutrition (SDG 2), preventing child mortality and decreasing the risk of non-communicable diseases (SDG 3), and supporting cognitive development and quality education (SDG 4).

Although extensive evidence supports the importance of breastfeeding for infant health and development, many infants worldwide do not have access to breastfeeding or their own mother’s milk because of maternal death, illness, or separation. Up to 40% of infants in neonatal intensive care units globally cannot access sufficient mother’s milk during during the first days or weeks of life.^2^ These vulnerable infants—mainly preterm neonates with low birthweight—are at greater risk of morbidity and mortality from severe digestive complications, infections, and delayed growth or development than full-term or healthy infants. For these infants, WHO recommends safe use of donor human milk through human milk banks as a key risk reduction strategy.^3^

To address this need, over 600 human milk banks have been established in more than 60 countries, but most are in Europe, the USA, Asia, and Brazil, with a nationalised system of more than 230 human milk banks in Brazil alone.^4^ More of these banks are needed. Human milk banks serve a critical role by recruiting milk donors, and collecting, processing, screening, storing, and distributing safe donor human milk. Without ensuring a strong global breastfeeding culture and support system, not all vulnerable infants will have access to donor human milk. Global scale-up of human milk banks has been hampered by the absence of global policies and standards to guide donor programmes. For example, there is no consensus on the classification of human milk as a food, tissue, medicine, or other possible classification for regulatory purposes, which has important implications for how human milk banks are governed and legislated. To date, in most countries, human milk banks are developing operating systems and guidelines without systematic coordination or with limited guidance on global best practices.

There are a number of important ethical considerations that policymakers, regulators, health workers, and mothers must consider in facilitating and navigating supportive breastfeeding environments, including the provision of human milk within different socioeconomic contexts.

Ethical challenges include the commercialisation of donor human milk for profit and exploitation of women in low-income settings selling breastmilk—where detrimental to their own child’s health—for purchase by consumers in high-income settings;^5^ managing risks associated with peer-to-peer sharing and selling of unscreened human milk;^6^ weighing trade-offs for increasing screening of donors to ensure milk safety with increasing supply to address unmet need;^7^ potential risks that donor’s own infants are not optimally breastfed; and determining how best to prioritise provision of donor human milk in times of scarcity.^8^ A crucial and overriding challenge is ensuring the appropriate use of donor milk so that it serves as a bridge to support breastfeeding, rather than displacing a mother’s own milk and breastfeeding.^9,10^

To address these challenges, the Ethox Centre of the University of Oxford and PATH convened a working group—the Oxford-PATH Human Milk Working Group— of technical and policy experts in nutrition, human milk banking, human rights, bioethics, and maternal, newborn, and child health to develop ethical guidance in support of safe and equitable access to human milk and breastfeeding for vulnerable infants. Participants shared perspectives from Australia, France, Germany, India, Kenya, Malawi, Mexico, the Philippines, South Africa, UK, USA, and Vietnam. The working group identified cross-cutting ethical considerations and key actions that should be addressed as part of global and regional responses to donor milk policy and guideline development (panel).

The working group calls on global, regional, and country-level policy and regulatory bodies to establish governance mechanisms and enact legislation for the safe and ethical use of donor human milk in a way that also protects, promotes, and supports breastfeeding— similar to the policies established to guide the safe, ethical use of medical products of human origin. These standards and policies are urgently needed to inform the development and operations of donor human milk programmes at the national, regional, and global levels. By heeding this call, we can work together to protect breastfeeding and safe, equitable access to human milk for vulnerable infants, and for all infants and young children globally.
